# Gender Differences in Family Caregiving. Do female caregivers do more or undertake different tasks?

**DOI:** 10.1186/s12913-024-11191-w

**Published:** 2024-06-14

**Authors:** Diana Pacheco Barzallo, Aline Schnyder, Claudia Zanini, Armin Gemperli

**Affiliations:** 1 Present Address: Faculty of Health Sciences and Medicine, University of Lucerne, Alpenquai 4, Lucerne, 6005 Switzerland; 2 Swiss Paraplegic Research, Guido A. Zäch Str. 4, Nottwil, 6207 Switzerland; 3 Center for Rehabilitation in Global Health Systems, WHO Collaborating Center, University of Lucerne, Frohburgstrasse 3, Lucerne, 6002 Switzerland; 4 Center for Primary and Community Care, Frohburgstrasse 3, 6002 Lucerne, Switzerland

**Keywords:** Family caregivers, Gender differences, Caregiving, Disability

## Abstract

**Background:**

Two out of three family caregivers are female. However, current trends show that men are more likely to undertake caregiving duties, yet female caregivers report a higher burden. This paper analyzed data from long-term family caregivers to determine whether, under similar circumstances, gender differences in caregiving persist. We examined whether the observed gender gap affects caregivers' satisfaction with their health and quality of life.

**Methods:**

We analyze cross-sectional data from family caregivers of persons with spinal cord injury (SCI) in Switzerland. The data provides comprehensive information about the time and type of weekly tasks family caregivers undertake. To determine differences in caregiving related to gender, we balanced the characteristics of the caregiver and the cared-for person using a propensity score kernel matching. With the balanced sample, we estimated how the observed differences in caregiving varied across cohorts using a Poisson regression.

**Results:**

Under similar circumstances, male and female caregivers invest similar time in caregiving. This result holds for 21 caregiving tasks, except for household chores, where women spent, on average, four more hours per week than male caregivers. Despite these differences, female caregivers report a quality of life and satisfaction with their health that is similar to that of male caregivers.

**Conclusion:**

Gender differences in caregiving narrow over time, except for household chores, where female caregivers continue to spend significantly more hours than male caregivers. Measures designed for family caregivers must consider these gender differences, as the support needs of female caregivers can differ greatly from those of male caregivers.

**Supplementary Information:**

The online version contains supplementary material available at 10.1186/s12913-024-11191-w.

## Introduction

Having a family caregiver is crucial for individuals facing disability, as it allows them to reside in their own homes instead of in institutional settings [1]. This arrangement, when desirable, holds immense value for the well-being of the cared-for person, as it enables them to benefit from a familiar environment and maintain their social connections, thus ensuring continuity in social participation [2]. However, health and social systems do not sufficiently acknowledge the work of family caregivers, even when their role is deemed essential to cope with increasing care needs in the population [3, 4]. In fact, due to the rise in the prevalence of chronic conditions, a big share of the population is expected to have a loss in intrinsic capacity, which translates into disability that requires care and support from others [5, 6]. Depending on the system’s organization and the family situation, care needs can be undertaken by professional care services (formal care) or by family, friends, or neighbors who are regularly unpaid (informal caregivers) [5].

Across OECD countries, one in ten individuals provides regular support and care to a relative, and two-thirds are female [7]. This disparity can be attributed to gender role socialization and the historical tendency for women to be less involved in the labor market, dedicating their time primarily to family responsibilities, such as childcare and household tasks [8]. However, over the past few decades, this situation has undergone some changes [9], with higher participation of women in the labor market and men more involved in caregiving tasks. Yet, even in cases where men and women share similar responsibilities in the household, the caregiving burden remains more prevalent among female caregivers [10–12].

Related literature has attempted to explain why female caregivers report a higher burden, with the obvious answer that even when household responsibilities are shared, women put many more hours into caregiving than men [13]. Nevertheless, when disaggregating the data, the difference between males and females is not appreciably wider and is narrowing over time [14–19]. Alternative hypotheses have arisen that posit that female caregivers experience caregiving differently from men or that female caregivers generally face additional stressors, such as financial constraints and more issues in combining caregiving tasks with other responsibilities, which may explain their higher burden [20, 21]. While both explanations are plausible, little evidence contrasts the differences in caregiving solely related to gender. The issue arises because caregiving is not homogeneous; the care needs of older persons are not the same as those of children or persons with different health conditions [22]. Thus, comparing the gender differences in caregiving is not simple, primarily due to the lack of data because caregiving remains organized in a private environment. Caregiving is, in fact, a heterogeneous process involving a range of tasks, and the effect on the caregiver can vary significantly according to the relationship to the cared-for person and whether the caregiving is short-term or long-term.

This paper addresses this gap in the literature by analyzing differences in caregiving solely related to gender. To do so, we analyzed what caregivers do and how much they do if they face similar circumstances, i.e., we compared male and female caregivers with similar characteristics (demographics, working status, and external support) and with similar demands for care. We also examined whether these differences explain gaps in health satisfaction and quality of life reported by family caregivers. In this study, we analyzed comprehensive data on caregivers of persons with spinal cord injury (SCI), which is an irreversible health condition that requires long-life support and care from others [23]. Caregiving in SCI is distinct from other health conditions as it is mostly caused by a sudden traumatic event, which leaves no possibility of anticipation to organize caregiving at home. This explains why persons with SCI are often younger individuals whose caregivers are typically their partners, and both are well below retirement age [23]. Caregiving for persons with SCI is demanding and of long-term; it involves a series of tasks that go from support for eating and drinking to paperwork and accompanying management of bodily wastes.

In Switzerland, long-term care (LTC) comprises all care services not provided in an inpatient hospital setting. These services are designed to meet a person’s health or care needs when they cannot perform everyday activities independently. LTC can be provided in skilled nursing homes or the person’s home. It is organized on a needs-based structure, where nursing care and assistance with activities of daily living are covered by mandatory insurances, with some cost sharing, if prescribed by a physician after a standardized care needs assessment [24]. Household support is not reimbursed by mandatory health insurance, which implies family members play a key role in supporting their relatives with shopping, accompaniment, paperwork, or other care tasks; however, these services can be covered by private insurances or out-of-pocket payments. In addition, and depending on the specific situation of the cared-for person, financial support is available to pay for care support, which must be undertaken by professional caregivers [24]. Due to the decentralized organization of LTC in the country, some Cantons provide tax deductions or special financial support to families with a relative in need of regular care; no direct financial support exists for family caregivers [25]. The latest estimates show that nearly 5% (around 400,000 people) of the Swiss population undertakes caregiving tasks for a relative [26, 27].

In this study, we examine how male and female relatives engage in caregiving to gain valuable insights and inform the development of targeted interventions. To enhance the planning and design of services aimed at relieving and supporting family caregivers, it is important to investigate gender differences in caregiving practices. This includes understanding whether female caregivers invest more hours in caregiving than male caregivers, examining the time devoted to various caregiving tasks by gender, and exploring whether male and female caregivers delegate the same activities to professional caregivers.

## Methodology

### Data

We analyzed primary data from a survey launched in 2016 and closed in 2017 on family caregivers of persons with SCI. We contacted persons with SCI living in the community using the registries from the Swiss Spinal Cord Injury Cohort Study (SwiSCI) [27]. The invitation asked the person with SCI to give the questionnaire to their primary informal caregiver to learn from the main care provider. If the persons with SCI did not have a caregiver, they were asked to return the questionnaire indicating so. Family caregivers could answer the questionnaire using a paper-pen document or online. If they required additional support to complete the questionnaire, they could do it via phone or face-to-face interview with one of our collaborators. Only caregivers over 18 who could answer the questionnaire in one of the three official Swiss languages—German, French, or Italian—were eligible to participate. The questionnaire was sent to 4,502 persons with SCI; however, those without a caregiver were excluded. A total of 717 informal caregivers participated in the study, a response rate of 35% [2].

The questionnaire was designed by a multidisciplinary team that included nurses, clinical SCI specialists, social workers, patient and homecare representatives, health sciences researchers, and people with SCI. It included 128 items using different tested instruments about socio-demographics, health services utilization, family arrangements, and social life, among other areas, extensively detailed in the survey protocol [23].

Family caregivers were asked to detail their caregiving responsibilities during a regular week. Caregivers were asked to report the total time (in hours) spent on caregiving and among 22 tasks. The tasks included activities of daily living (ADL), e.g., toileting, dressing, transferring or ambulating, and instrumental activities of daily living (IADL), e.g., shopping, cooking, housekeeping. A complete list of the tasks is detailed in Appendix 1. Finally, family caregivers were asked about other sources of support they receive, such as other relatives and professional home care.

### Statistical analysis

To determine differences in caregiving solely related to gender, it was necessary to compare male and female caregivers in a similar setting, i.e., caregivers of similar characteristics and with similar care responsibilities. However, as with any other health condition, caregivers of persons with SCI are more likely to be female not only because SCI has a higher incidence among males but also because women are more likely to take care of responsibilities in a household. Thus, a simple comparison of caregiving duties between genders would not account for the difference in gender distribution.

To compare how caregiving varies between genders, we implemented a propensity score matching (PSM) to balance the characteristics of male and female caregivers in the sample. PSM included characteristics of the caregiver: age, relationship with the cared-for person—employment status, educational level, family income, and whether they received external support in the caregiving tasks. The matching also included the characteristics of the cared-for person to account for the care needs. It included the age of the persons with SCI, injury level, and dependency level to account for the effort required by family caregivers to care for them. In addition, as SCI is mostly caused by a traumatic event, family members of persons with SCI had no anticipation of organizing their lives around caregiving needs, so the caregiving role can be considered a random event.

The balancing implemented an Epanechnikov kernel with a 0.1 bandwidth, where male caregivers are weighted as a function of the distance to female caregivers. Closer caregivers have similar characteristics and thus are weighted higher in the comparison. Less similar caregivers have a smaller weight in the comparison. With the balanced sample, we compared caregiving tasks and time investment. To define how large the differences between groups are, we calculated standardized differences, where an absolute size of 0.2 is considered a small difference, 0.5 is a medium difference, and 0.8 is a large difference [28].

#### Cohort effects

Finally, we analyzed cohort effects to determine if there is a shift in the involvement of male relatives in caregiving. Using a Poisson regression, we predicted how much time male caregivers devoted to household chores by age. If there is a cohort effect, we expect that the gender gap in household chores narrows with younger generations of caregivers.

## Results

### Descriptive data

Table 1 reports the main characteristics of the family caregivers by gender. The total sample had data from 714 caregivers, of whom 72% were female, which aligns with the higher incidence of SCI among men [29]. Some differences were observed between genders—male caregivers were, on average, older and had more years of education than female caregivers: 40% of male caregivers were over 65, while only 29% of female caregivers were in the same age range. Similarly, most caregivers in the sample had a middle-level education, but male caregivers were likelier to have tertiary education than female caregivers (28% vs. 24%). On average, family caregivers spent 21.5 hours per week on caregiving tasks, with no marked difference between genders, and almost half of the participants reported being caregivers for more than 10 years.

Regarding labor market participation, close to 50% of the sample reported being involved in a paid activity. These numbers showed a difference between the genders. While a large proportion of male caregivers (27.6%) worked full-time jobs, only 6.8% of female caregivers had full-time jobs. This number is reversed for part-time jobs, in which 33% of female caregivers had part-time jobs compared to 10% of male caregivers. Another difference is the number of people who reported being homemakers: male homemakers were 2% compared to 13% female homemakers. Due to age differences, we also observed more retired male caregivers. Male caregivers had almost twice the unemployment rate of female caregivers. Regarding the financial situation, half of the sample lived in households with incomes above the median income in the country [30, 31]. However, female caregivers were likelier to live in households with lower incomes than male caregivers.

Most caregivers cared for their partner or spouse, and close to one-third of the sample reported not receiving additional support for caregiving. Approximately 25% of the sample received professional home care support, regularly organized to cover specific tasks. While female caregivers received relatively more support from family members, male caregivers received more support from professional care services.

Finally, caregivers in our sample reported high satisfaction with their health, 7.4 on a 1 to 10 scale. More than 75% of the sample reported a good or very good quality of life. Male caregivers, however, were likelier to report having a bad or very bad quality of life than female caregivers.

**Table 1:** Characteristics of the family caregivers

	**Sex of the family caregiver**		**Total sample**
	**Male**	**Female**	
	*N* = 203	*N* = 511	*N* = 714
**Age in years** – mean (SD)	59.1 (14.6)	56.5 (13.6)	57.2 (13.9)
**Age group**			
18—35	16 (7.9%)	42 (8.2%)	58 (8.1%)
36—54	54 (26.6%)	170 (33.3%)	224 (31.4%)
55—65	52 (25.6%)	151 (29.5%)	203 (28.4%)
65 +	81 (39.9%)	148 (29.0%)	229 (32.1%)
**Caregiving hours/week** – mean (SD)	21.3 (24.1)	21.3 (24.5)	21.5 (24.9)
**Years as caregiver**			
< 1 year	20 (9.9%)	40 (7.8%)	60 (8.4%)
1–2 years	16 (7.9%)	26 (5.1%)	42 (5.9%)
2–5 years	33 (16.3%)	93 (18.2%)	126 (17.6%)
5–10 years	40 (19.7%)	108 (21.1%)	148 (20.7%)
> 10 years	94 (46.3%)	244 (47.7%)	338 (47.3%)
**Education level**			
No Education	9 (4.4%)	20 (4.0%)	29 (4.2%)
Compulsory school	51 (25.1%)	126 (25.0%)	177 (25.4%)
High school/vocational school	76 (37.4%)	237 (47.1%)	313 (45.0%)
Tertiary and higher	57 (28.1%)	120 (23.9%)	177 (25.4%)
**Working Status**			
Full-time	56 (27.6%)	35 (6.8%)	91 (12.7%)
Part-time	20 (9.9%)	168 (32.9%)	188 (26.3%)
In education	2 (1.9%)	9 (1.8%)	11 (1.5%)
Unemployed	11 (5.4%)	15 (2.9%)	26 (3.6%)
Works in family business	1 (0.5%)	21 (4.1%)	22 (5.0%)
Protected job	4 (2.0%)	3 (0.6%)	7 (3.1%)
Homemaker	4 (2.0%)	68 (13.3%)	72 (10.1%)
Retired due to age	3 (1.5%)	1 (0.2%)	4 (0.6%)
Other activity	2 (1.0%)	16 (3.1%)	18 (2.5%)
**Household Income**			
< 1500 CHF	1 (0.5%)	2 (0.4%)	3 (0.5%)
1500–3000 CHF	18 (9.8%)	31 (6.6%)	49 (7.5%)
3000–4500 CHF	24 (13.1%)	78 (16.7%)	102 (15.7%)
4500–6000 CHF	43 (23.5%)	98 (20.9%)	141 (21.7%)
6000–7500 CHF	22 (12.0%)	95 (20.3%)	117 (18.0%)
7500–9000 CHF	28 (15.3%)	66 (14.1%)	94 (14.4%)
> 9000 CHF	47 (25.7%)	98 (20.9%)	145 (22.3%)
**Children in household**	2 (0.1%)	9 (1.8%)	11 (4.7%)
**Marital status**			
Married/partner	157 (77.3%)	401 (78.5%)	558 (78.2%)
No partnership	39 (19.2%)	105 (20.5%)	144 (20.2%)
**Relationship with cared-for person**			
Spouse/life partner	144 (70.9%)	393 (77.0%)	537 (75.1%)
Child	13 (6.4%)	25 (4.9%)	38 (5.3%)
Sibling	6 (3.0%)	15 (2.9%)	21 (2.9%)
Mother/father	22 (10.8%)	72 (14.1%)	94 (13.2%)
Other relatives	5 (2.5%)	3 (0.6%)	8 (1.1%)
**Has no external support**	58 (28.6%)	156 (30.5%)	214 (30.0%)
**Has external support from**			
Relatives	37 (18.2%)	99 (19.4%)	136 (19.0%)
Friends and others	37 (18.2%)	84 (16.4%)	121 (16.9%)
Professional support	71 (35.0%)	172 (33.7%)	243 (34.2%)
**Health satisfaction**^**a**^ – mean (SD)	7.2 (2.2)	7.5 (2.1)	7.4 (2.2)
**Quality of life**			
Very good	40 (19.7%)	118 (23.1%)	158 (22.1%)
Good	113 (55.7%)	280 (54.8%)	393 (55.0%)
Neither nor	39 (19.2%)	94 (18.4%)	133 (18.6%)
Bad	5 (2.5%)	5 (1.0%)	10 (1.4%)
Very bad	1 (0.5%)	2 (0.4%)	3 (0.4%)

*SD* Standard deviation

^a^Scale from 1 (worst) to 10 (best)

Table 2 summarizes the characteristics of the cared-for person by the gender of the family caregiver. On average, the cared-for person was 56 years old. As most caregivers cared for their partners, male caregivers mostly cared for females with SCI, while female caregivers cared for males with SCI. Interestingly, on average, female caregivers supported and cared for persons with higher needs than male caregivers: female caregivers looked after people with tetraplegia who were dependent on their wheelchairs and whose injury was the result of an accident.

**Table 2:** Characteristics of the cared-for person

	**Sex of the family caregiver**		**Total sample**
	**Male**	**Female**	
	*N* = 203	*N* = 511	*N* = 714
**Age in years** – mean (SD)	56.4 (15.8)	56.4 (16.5)	56.4 (16.3)
**Sex**			
Male	58 (28.5%)	459 (89.8%)	517 (72.4%)
Female	135 (66.5%)	47 (9.2%)	182 (25.5%)
**Injury type**			
Paraplegia	131 (64.5%)	300 (58.7%)	431 (60.4%)
Tetraplegia	49 (24.1%)	174 (34.1%)	223 (31.2%)
**Cause of the injury**			
Due to an accident	111 (54.7%)	396 (77.5%)	507 (71.0%)
Due to sickness	55 (27.1%)	69 (13.5%)	124 (17.4%)
Other causes	25 (12.3%)	33 (6.5%)	58 (8.1%)
**Dependency level**			
Wheelchair	116 (57.1%)	365 (71.4%)	481 (67.4%)
Able to stand	10 (4.9%)	13 (2.5%)	23 (3.2%)
Ability to walk	63 (31.0%)	106 (20.7%)	169 (23.7%)

*SD* Standard deviation

### Statistical analysis

Table 3 reports the results of propensity score matching. The table is divided into two sections, one showing the characteristics used to balance the sample and the second showing the results in the outcomes of interest. In addition, we report the unmatched and matched samples to illustrate the size of the adjustment to reach comparable groups in the sample. See Appendix 2 for the distribution of matched and unmatched samples. Although matching did a good job, small differences persisted between the groups. Compared to male caregivers, female caregivers were, on average, likelier to be employed in part-time jobs and had spent more years as caregivers.

**Table 3:** Unmatched and matched sample. results reported by adjusted characteristics and outcomes

	**Unmatched sample** *N* = 717		**Matched sample (Kernel)** *N* = 528			
	**Male**	**Female**	**Male**	**Female**	**Difference**	**St. Difference**
**Matching characteristics**						
Age	59.1	56.5	55.9	55.9	-0.03	0.18
In partnership	0.71	0.77	0.77	0.77	0.01	-0.14
Works	0.40	0.44	0.45	0.47	-0.02	-0.09
**Works part-time**	0.15	0.43	**0.43**	**0.46**	**-0.02**	**-0.57**^**b**^
Tertiary education	0.30	0.24	0.26	0.26	0.00	0.13
**Years as caregiver**	11.4	13.6	**12.1**	**13.0**	**-0.91**	**-0.22**^**a**^
Does not have external support	0.29	0.31	0.31	0.31	0.00	-0.04
Household income	4.85	4.80	4.65	4.86	-0.21	0.03
Age cared-for person	56.4	56.4	56.8	55.8	1.03	0.00
Paraplegia	0.65	0.59	0.59	0.59	0.00	0.12
**Dependent on wheelchair**	0.57	0.71	**0.73**	**0.72**	**0.01**	**-0.32**^**a**^
**Comparison outcomes**						
**Caregiving hours by task**						
Eating and drinking	2.04	1.78	2.22	1.86	0.36	0.05
Wash face	0.84	0.67	1.05	0.73	0.32	0.05
Wash upper-body	0.59	0.49	0.83	0.52	0.31	0.04
Wash feet	0.66	0.51	0.82	0.59	0.23	0.06
Wash lower-body	0.49	0.67	0.72	0.73	-0.01	-0.10
Dress upper-body	0.73	0.69	1.22	0.75	0.47	0.01
Dress lower-body	0.77	0.91	0.97	0.99	-0.02	-0.06
Respiratory care	0.24	0.26	0.37	0.24	0.13	-0.01
Bladder mgmt	0.83	0.66	1.22	0.65	0.56	0.06
Bowel mgmt	0.68	0.73	1.40	0.78	0.62	-0.02
Transfer to bed	1.01	0.66	0.95	0.68	0.27	0.17
Transfer to bathtub	0.42	0.33	0.45	0.37	0.08	0.06
Climb stairs	0.32	0.09	0.41	0.08	0.33	0.10
Move in the house	0.58	0.24	0.75	0.25	0.50	0.12
Car transfer	0.54	0.56	0.79	0.56	0.23	-0.01
Move outdoors moderate dis	0.68	0.39	0.82	0.44	0.37	0.13
Move outdoors long dis	1.35	0.83	1.64	0.95	0.69	0.14
Accompanying	1.11	0.89	1.34	0.93	0.41	0.09
** Housekeeping**	5.33	9.73	**6.60**	**10.43**	**-3.84**	**-0.45**^**a**^
Shopping	2.37	2.25	2.59	2.33	0.26	0.04
Paperwork	0.97	0.83	1.02	0.88	0.14	0.06
Others	1.01	0.95	1.54	0.96	0.58	0.02
**Total caregiving hour**s	21.3	21.3	23.0	21.2	1.77	0.00
**Satisfaction with health**	7.18	7.45	7.39	7.48	-0.09	-0.13
**Quality of life**	2.94	3.02	3.00	3.04	-0.03	-0.11

^1^Standardized differences: ^a ^small if equal to >=0.2; ^b^ medium if >=0.5; *** big if >=0.8

**Fig. 1:**
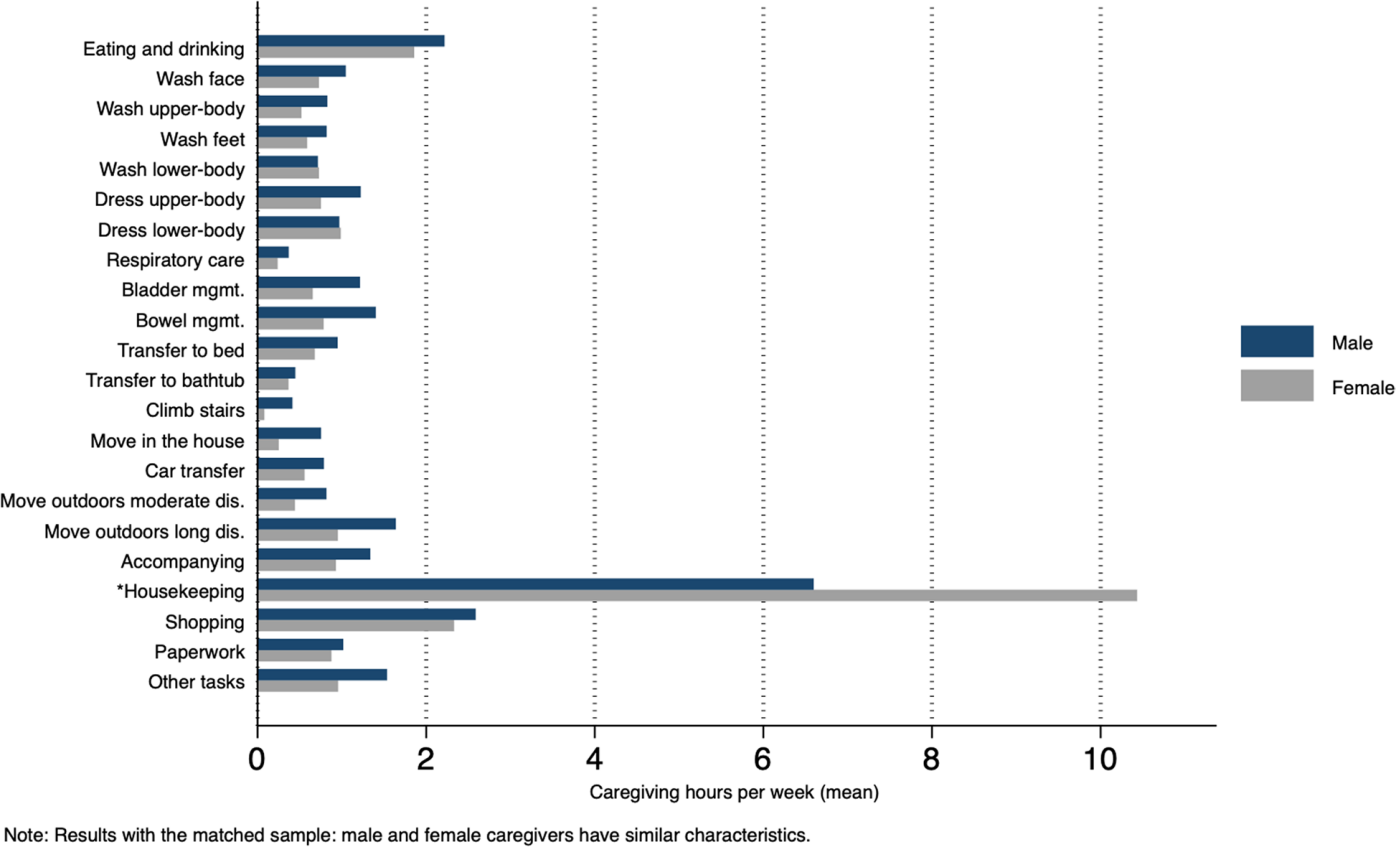
Differences in caregiving tasks (hours) between genders

Regarding outcomes, total hours of caregiving were not significantly different between the genders. Disaggregating the caregiving hours by task (Fig. 1) reveals that, on average, male and female caregivers performed similar activities. There were no marked differences in the hours spent on caregiving tasks, except for household chores, on which women spent 10.4 h and men spent 6.6 h per week. Within the matched sample, male and female caregivers reported similar life satisfaction and quality of life.

#### Cohort effects

**Fig. 2:**
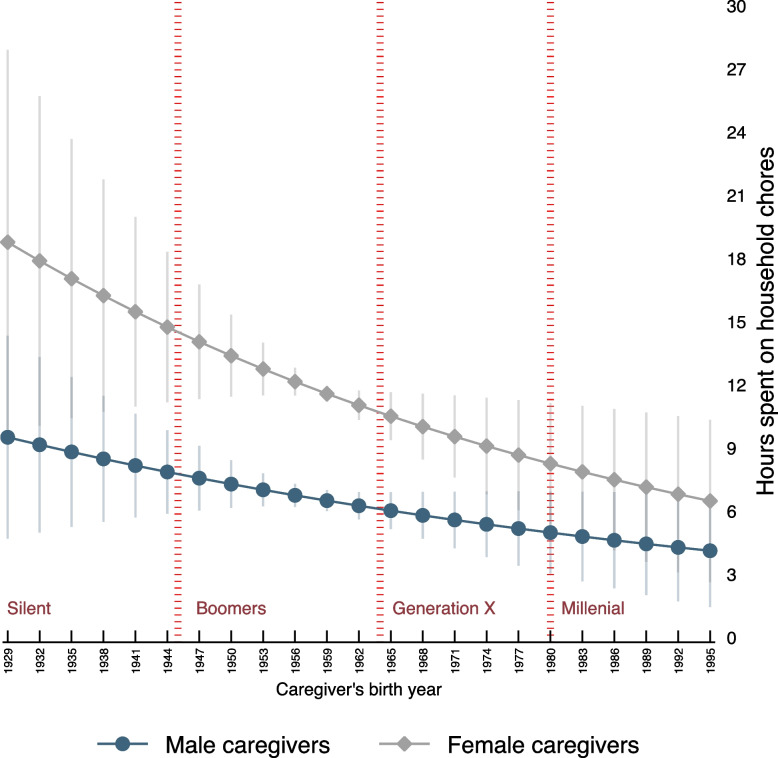
Cohort effects—Time spent on household chores across generations

Finally, we disaggregated the results observed in household chores to see how gender differences change across generations. The results are presented in Fig. 2, which displays the predicted hours caregivers spend on household chores by the caregiver's birth year. On average, male and female caregivers from older generations (silent and boomer) spend more time on caregiving than younger generations; yet, the time spent by female caregivers on household chores is three times that of male caregivers. The observed gap narrows with younger generations, where millennial male and female caregivers spend comparable time on household chores. Interestingly, the reduction in the gap is explained by younger female caregivers investing significantly fewer hours in household chores and not by male caregivers taking over a larger share of this task.

## Discussion

Our findings challenge conventional assumptions regarding the distribution of caregiving responsibilities between male and female caregivers. Under similar circumstances, our results indicate no marked differences in the overall time spent on caregiving tasks between male and female caregivers, except for household chores. On average, women spent 60% more time on household chores than their male counterparts (10.4 h for women vs. 6.6 h for men). Yet, female caregivers report similar levels of satisfaction with their health and quality of life to male caregivers. Interestingly, the observed gap in household chores narrows with younger generations, mainly because female caregivers undertake fewer household chores and not because male caregivers undertake a larger share of the task.

These findings align with recent studies showing a declining trend in gender differences in caregiving, yet more traditional views seem to prevail when caregiving responsibilities involve household chores [13, 19]. The gap in household chores is also observed in related studies done for the general population, where females always do more housework than males, even in contexts where they do not have to care for other persons [11, 12]. Since females are still the primary source of informal caregiving worldwide, it is important to understand why gender differences prevail in household chores. Are the observed differences explained by individual preferences, external constraints, or social conditioning related to gender roles in which women are left with the least preferred tasks?

An additional plausible explanation of our results is that, even in similar contexts, male caregivers have a better financial situation than female caregivers (gender pay gap), mostly because men are more likely to be employed in higher-paying jobs [32, 33]. Thus, male caregivers are likelier to have the financial resources to pay for external help and free themselves from the least preferred tasks, like household chores. In contrast, female caregivers face more financial constraints and are more likely to be involved in lower-paying jobs, making them less likely to pay for external help. This difference would explain why female caregivers end up with more household chores and also receive more help from relatives than from professional sources.

Further research is needed to explore the underlying factors contributing to observed gender differences in caregiving, including the influence of social, cultural, and contextual factors [34]. Although our results are based on a large sample of long-term, highly burdened family caregivers, it is important to acknowledge that the observed gender differences come from a sample in which, in most cases, male caregivers care for a female and female caregivers care for a male. Following our results, caring for a male or a female may not be interchangeable, as expectations, needs, and social norms may vary between genders. In fact, related literature has shown how gender differences in caregiving affect women, who generally undertake many more household chores once married, a situation that worsens with children in the household. In contrast, men tend to save some time doing household chores once married [35].

Informal caregivers are the pillar of long-term care, yet most of what they do goes unrecognized [25]. Without family caregivers, the increasing need for care in the population can put the sustainability of health and social systems at risk [1, 36]. Thus, to guarantee the involvement of relatives in the caregiving process, governments should foster collaborations between healthcare providers, social services, and community organizations to ensure coordinated support for family caregivers, especially in demanding contexts like caregiving for persons with SCI [37]. For example, measures to support family members in combining their caregiving responsibilities with their professional careers can effectively reduce gender differences [38–40]. More flexibility from employers can be very effective in supporting employees with caregiving responsibilities [38]. Along the same lines, for caregivers with more financial constraints, financial assistance programs (e.g., caregiver stipends or tax benefits) can help alleviate their financial burden, especially by considering how much their work saves health and social systems [41]. Third, considering that 40% of male caregivers are retired in our sample, policies should also address the issue of caregiving and aging, for instance, by introducing initiatives to promote the physical and mental health of older caregivers [42].

## Conclusion

Females in the household are more likely to become caregivers in all care needs contexts. However, if a male relative becomes a caregiver, he undertakes similar tasks and spends a similar amount of time caregiving, except for household chores. Interestingly, female caregivers reported a similar quality of life and satisfaction with their health to that of male caregivers. Understanding what caregivers do and how much they do is essential to finding ways to support their role; however, gender differences must be considered, as what female caregivers need may differ from what male caregivers need.

### Supplementary Information

Supplementary Material 1.

Supplementary Material 2.

## Data Availability

The data that support this study's findings are available from Swiss Paraplegic Research (SPF), but restrictions apply to their availability. These data were used under license for the current study and are not publicly available. However, data are available from the corresponding author upon reasonable request and with permission of SPF.
