# Supplementary material for: Gender Differences in Family Caregiving. Do female caregivers do more or undertake different tasks?
Source: BMC Health Serv Res. 2024 Jun 14;24:730. doi: 10.1186/s12913-024-11191-w (PMC11177503; doi:10.1186/s12913-024-11191-w)
Supplement: Supplementary file 1 — Supplementary Material 1. [file 12913_2024_11191_MOESM1_ESM.pdf]

Appendix 1: Informal Caregivers Questionnaire – English version

Items related to caregiving investment

Investment in Caregiving

This section is about your situation as a caregiver. We would like to learn from you how much you invest, what tasks you fulfil and what barriers you perceive.

29. Please indicate the year in which you first took over the caregiving

✎............... (such as: 2011)

30. Were there any noteworthy interruptions since then?

○ No to Q32

○ Yes.

How many month in total.

✎............... (number of months)

31. Please indicate the reasons for the interruptions

✎...............

32ff **In what activities do you help the person with SCI and how many hours do you spend every week on them?**

Please indicate how many hours you spent per week.

Activities	Numbers of hours per week	Not applicable
**32. Eating and drinking**	✎...............	○
**33. Washing face and hands**	✎...............	○
Please think about your assistance in hand washing, face washing, tooth brushing, combing, shaving or make-up		
**34. Washing upper body and head**	✎...............	○
**35. Washing feet**	✎...............	○
**36. Washing lower body**	✎...............	○
**37. Dressing upper body**	✎...............	○
Dressing the upper body includes putting on and taking off clothes like t-shirts, blouses, shirts, bras, shawls, or orthoses (e.g., arm splint, neck brace, and corset).		
**38. Dressing lower body**	✎...............	○
Dressing the lower body includes putting on and taking off clothes like shorts, trousers, shoes, socks, belts, or orthoses (e.g., leg splint).		
**39. Caregiving in relation to Respiration**	✎...............	○
**40. Bladder management and use of toilet**	✎...............	○
**41. Bowel management and use of toilet**	✎...............	○
**42. Transfer between bed and wheelchair**	✎...............	○
**43. Transfers from the wheelchair to the toilet/tub**	✎...............	○
**44. Climbing stairs**	✎...............	○

45. **Moving indoors** ✐............... ○

46. **Transfer between wheelchair and car** ✐............... ○

47. **Moving outdoors moderate distance (10 to 100 meter)** ✐............... ○

48. **Moving outdoors over 100 meter** ✐............... ○

49. **Company to therapy, doctor visits, and to other offices** ✐............... ○

50. **Housekeeping (cooking, tidying, laundry etc)** ✐............... ○

51. **(Grocery) shopping** ✐............... ○

52. **Paperwork (fill out forms, handling bills, bank or financial affairs)** ✐............... ○

53. **Others, please specify** ✐...............

✐...............

54. How many hours in total do you spend on the caregiving?

✐............... (number of hours per week)

55. Who else takes care of the person with spinal cord injury in the daily living?

Check all that apply

- ☐ Spouse/partner of the person
- ☐ Child(ren) of the person
- ☐ Parent(s) of the person
- ☐ Sibling(s) of the person
- ☐ Friend(s)/acquaintance(s)/neighbor(s) of the person
- ☐ Professional home care, hours per week ✐...............
- ☐ Other persons, namely ✐...............
- ☐ Other institutes ✐...............
