# Supplementary figures and images for: Gender Differences in Family Caregiving. Do female caregivers do more or undertake different tasks?
Source: BMC Health Serv Res. 2024 Jun 14;24:730. doi: 10.1186/s12913-024-11191-w (PMC11177503; doi:10.1186/s12913-024-11191-w)

**Appendix 2:** Figure: Distribution of the matched and unmatched samples

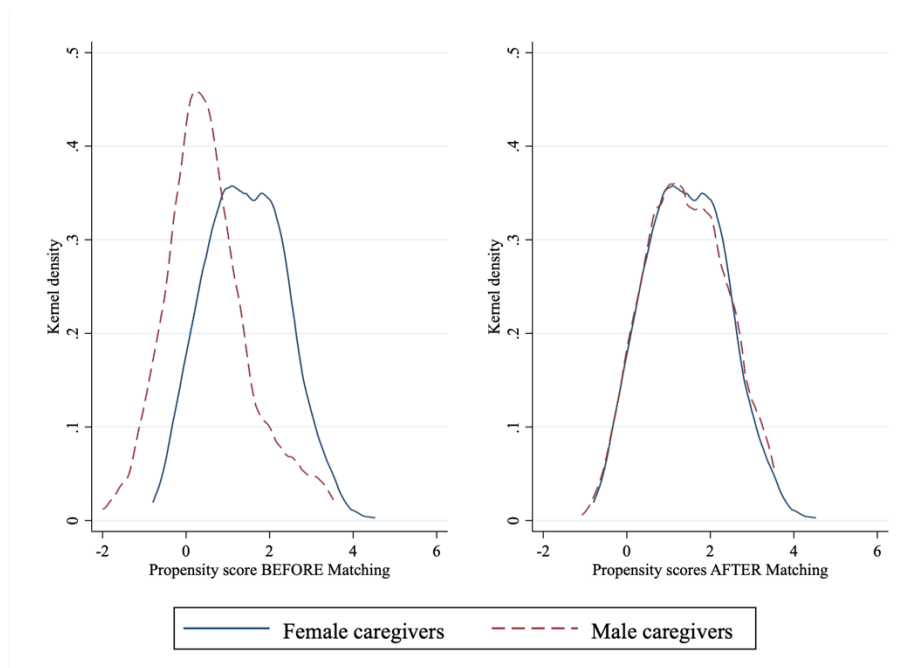

Supplement: Supplementary file 2 — Supplementary Material 2. [file 12913_2024_11191_MOESM2_ESM.pdf]
